# Whole-exome analysis of 177 pediatric patients with undiagnosed diseases

**DOI:** 10.1038/s41598-022-14161-6

**Published:** 2022-08-26

**Authors:** Kotaro Narita, Hideki Muramatsu, Satoshi Narumi, Yuji Nakamura, Yusuke Okuno, Kyogo Suzuki, Motoharu Hamada, Naoya Yamaguchi, Atsushi Suzuki, Yosuke Nishio, Anna Shiraki, Ayako Yamamori, Yusuke Tsumura, Fumi Sawamura, Masahiro Kawaguchi, Manabu Wakamatsu, Shinsuke Kataoka, Kohji Kato, Hideyuki Asada, Tetsuo Kubota, Yukako Muramatsu, Hiroyuki Kidokoro, Jun Natsume, Seiji Mizuno, Tomohiko Nakata, Hidehito Inagaki, Naoko Ishihara, Takahiro Yonekawa, Akihisa Okumura, Tomoo Ogi, Seiji Kojima, Tadashi Kaname, Tomonobu Hasegawa, Shinji Saitoh, Yoshiyuki Takahashi

**Affiliations:** 1 Department of Pediatrics, Nagoya University Graduate School of Medicine, 65 Tsurumai-cho, Showa-ku, Nagoya, 466-8550 Japan; 2 Department of Molecular Endocrinology, National Research Institute for Child Health, Tokyo, Japan; 3 Department of Pediatrics, Keio University School of Medicine, Tokyo, Japan; 4 Department of Pediatrics and Neonatology, Nagoya City University Graduate School of Medical Sciences, 1 Kawasumi, Mizuho-cho, Mizuho-ku, Nagoya, 467-8601 Japan; 5 Medical Genomics Center, Nagoya University Hospital, Nagoya, Japan; 6 Department of Virology, Nagoya City University Graduate School of Medical Sciences, Nagoya, Japan; 7 Department of Genetics, Research Institute of Environmental Medicine, Nagoya University, Nagoya, Japan; 8 Department of Pediatrics, Japanese Red Cross Aichi Medical Center Nagoya Daiichi Hospital, Nagoya, Japan; 9 Department of Pediatrics, Anjo Kosei Hospital, Anjo, Japan; 10 Department of Clinical Genetics, Aichi Developmental Disability Center Central Hospital, Kasugai, Japan; 11 Division of Molecular Genetics, Institute for Comprehensive Medical Science, Fujita Health University, Toyoake, Japan; 12 Department of Pediatrics, Fujita Health University School of Medicine, Toyoake, Japan; 13 Department of Pediatrics, Mie University Graduate School of Medicine, Tsu, Japan; 14 Department of Pediatrics, Aichi Medical University, Nagakute, Japan; 15 Department of Genome Medicine, National Center for Child Health and Development, Tokyo, Japan

**Keywords:** Genetics research, Disease genetics

## Abstract

Recently, whole-exome sequencing (WES) has been used for genetic diagnoses of patients who remain otherwise undiagnosed. WES was performed in 177 Japanese patients with undiagnosed conditions who were referred to the Tokai regional branch of the Initiative on Rare and Undiagnosed Diseases (IRUD) (TOKAI-IRUD). This study included only patients who had not previously received genome-wide testing. Review meetings with specialists in various medical fields were held to evaluate the genetic diagnosis in each case, which was based on the guidelines of the American College of Medical Genetics and Genomics. WES identified diagnostic single-nucleotide variants in 66 patients and copy number variants (CNVs) in 11 patients. Additionally, a patient was diagnosed with Angelman syndrome with a complex clinical phenotype upon detection of a paternally derived uniparental disomy (UPD) [upd(15)pat] wherein the patient carried a homozygous *DUOX2* p.E520D variant in the UPD region. Functional analysis confirmed that this *DUOX2* variant was a loss-of-function missense substitution and the primary cause of congenital hypothyroidism. A significantly higher proportion of genetic diagnoses was achieved compared to previous reports (44%, 78/177 vs. 24–35%, respectively), probably due to detailed discussions and the higher rate of CNV detection.

## Introduction

Recently, whole-exome sequencing (WES), a next-generation sequencing method that selectively sequences protein-coding regions^1^, has been used for establishing genetic diagnoses among patients who remain otherwise undiagnosed. WES is a definitively cost-effective and powerful tool that can detect both single-nucleotide variants (SNVs) and very short (< 50 bp) insertions/deletions, and recent computational advances provide opportunities for the identification of larger copy number variants (CNVs)^2,3^ and uniparental disomy (UPD)^4^.

The Initiative on Rare and Undiagnosed Diseases (IRUD), led and coordinated by the Japan Agency for Medical Research and Development, was initiated in 2015 to accelerate the pioneering efforts made by an international collaboration among the Undiagnosed Diseases Program/Network in the United States^5,6^, the Finding of Rare Disease Genes program in Canada^7^, and the Deciphering Developmental Disorders in the United Kingdom^8^. As the IRUD regional headquarters office in the Tokai-area of Japan (TOKAI-IRUD), we held review meetings to conduct detailed analyses of each patient and to discuss cases with the doctors in-charge, genetic specialists, and specialists in pertinent medical fields. Here, we report the results of SNV, CNV, and UPD analyses of WES data from 177 patients with undiagnosed diseases registered in the TOKAI-IRUD program.

## Patients and methods

### Patients

The study cohort consisted of 177 undiagnosed Japanese patients with a median (range) age of 4 (0–30) years who were referred to the TOKAI-IRUD program. The patients and their family members gave written informed consent for genetic testing and publication of identifying information/images in an online open-access publication. The study protocol was approved by the Ethics Committee of the Nagoya University Graduate School of Medicine. Patients without suspected or known disease, who had not previously undergone genome-wide tests and fulfilled the following criteria for IRUD, as described below, were eligible for enrollment in this study^9^:

1. The patient remains undiagnosed for 6 months or longer (not necessary for infants) and the symptom(s) affects his/her daily life; AND,
2. There exists an objective sign(s) that cannot be traced to a single organ; OR.
3. There is a distinct family history suggestive of a genetic etiology (similar symptom(s) found in the patient’s relatives).

### Whole-exome sequencing

All methods were carried out in accordance with relevant guidelines and regulations. Genomic DNA was extracted from peripheral blood mononuclear cells using the QIAamp DNA Blood Mini Kit (Qiagen, Hilden, Germany). Trio-WES (patient, father, and mother) for 168 patients and duo-WES (patient and mother) in nine cases was performed using the SureSelect Human All Exon V6 kit for capture (Agilent Technologies, Santa Clara, CA, USA) and a HiSeq2500 system (Illumina, Inc., San Diego, CA, USA) for sequencing 101-bp paired-end reads. Obtained reads were aligned to the hg19 reference genome using the Burrows–Wheeler aligner (BWA, http://bio-bwa.sourceforge.net/) with default parameters and a –mem option. Polymerase chain reaction duplicates were removed using Picard tools (http://broadinstitute.github.io/picard/). Sequence variations were detected and annotated using VarScan2^10^ and ANNOVA R^11^, respectively. For germline variations, we removed common single-nucleotide polymorphisms (SNPs) (defined as those with > 1% allele frequency) using ExAC (http://exac.broadinstitute.org/), gnomAD (https://gnomad.broadinstitute.org/), 1000 genomes (http://www.1000genomes.org/), ESP6500 (http://evs.gs.washington.edu/EVS/), and an in-house database. Conclusive assessment of molecular variants was performed according to guidelines issued by the American College of Medical Genetics and Genomics (ACMG)^12^. InterVar (https://wintervar.wglab.org) was utilized to assist in the evaluation of variants, and conclusive evaluations were preformed after discussion in the review meetings. Among the variants defined as “pathogenic” or “likely pathogenic,” those considered to be concordant with medical histories and phenotypes were deemed “diagnostic.” Gene variants that are not known to be causative of human genetic diseases were not considered diagnostic in this analysis. None of the variants annotated as “pathogenic” or “likely pathogenic” were validated by Sanger sequencing.

Copy number analysis was performed using WES, as described previously^13^. Briefly, the coverage of each exon, normalized by the mean coverage of the entire sample, was compared with that of 12 unrelated reference samples. Exons exhibiting normalized coverage greater than three standard deviations of the reference samples were determined to be candidates for copy number alterations. All candidate exons were visually inspected using the Integrative Genomics Viewer^14^. CNVs larger than 1 Mb were evaluated according to ACMG guidelines^15^. Among the variants defined as “pathogenic” or “likely pathogenic,” those considered to be concordant with medical histories and phenotypes were deemed “diagnostic.”

Region of homozygosity (ROH) analysis was performed using H3M2 with recommended parameters (DNorm = 100,000, P1 = 0.1, P2 = 0.1)^16^. This is a discrete state Hidden Markov Model that includes a parameter to account for sequencing and alignment errors, as well as a parameter within the transition probabilities matrix to account for the inconsistent distance between SNPs within WES. The algorithm inputs a BAM file and calculates the ratio between alternate allele reads and total coverage at each polymorphic position (based on the 1000 Genomes Project). This ratio is used to determine the genotype state at each SNP. Long (> 10 Mb) stretches of homozygosity were deemed as potentially diagnostic UPD regions.

Patients and their guardians were informed of incidental findings in accordance with ACMG recommendations for reporting incidental findings during clinical exome and genome sequencing^17^.

### Functional analysis of E520D DUOX2

Expression vectors encoding hemagglutinin (HA)-tagged DUOX2 (HA-DUOX2) and FLAG-tagged DUOXA2 (DUOXA2-FLAG) have been described previously^18^. The E520D missense substitution was introduced by site-directed mutagenesis using the QuickChange XL Site-Direct Mutagenesis kit (Agilent Technologies). Human embryonic kidney (HEK) 293 cells were maintained in Dulbecco’s modified Eagle’s medium supplemented with 50 U/ml of penicillin, 50 g/ml of streptomycin, and 10% fetal bovine serum. To create HEK293 cells that stably express C-terminal FLAG-tagged DUOXA2 (DUOXA2-FLAG), the cDNA sequence of the human DUOXA2, along with the FLAG tag, were cloned into pB510B-1 (System Biosciences, Palo Alto, CA, USA). Stable transformants were established according to manufacturer’s protocol. Each DUOX2-expressing vector (DUOX2 wild type [WT] or E520D) was transiently transfected with Lipofectamine 3000 (Life Technologies, Carlsbad, CA, USA).

To evaluate the H_2_O_2_ production of each DUOX2 protein (WT or E520D), HEK293 cells were seeded into a 12-well plate and transfected with 1000 ng of HA-DUOX2. Cells were harvested at 48 h after transfection, washed with phosphate-buffered saline (PBS), and resuspended in 100 μl of Earle’s balanced salt solution (Sigma-Aldrich Corporation, St. Louis, MO, USA). Extracellular H_2_O_2_ production in the presence of 1 μM ionomycin (Sigma-Aldrich Corporation) was measured in cells expressing WT or mutant HA-DUOX2 that were resuspended in solution with Amplex Red reagent (Life Technologies), as per manufacturer’s instructions. The activity of the mutant was expressed as percentage (mean ± standard error) of WT activity. Background activity, measured in mock-transfected cells, was set to 0%. Data are representative of three independent experiments (each performed in triplicate) with similar results. Welch’s *t*-test was used for comparing H_2_O_2_ production.

For surface staining of HA-DUOX2 and DUOXA2-FLAG (WT or E520D), transfected cells were washed in PBS, fixed in 4% paraformaldehyde, and incubated with the anti-HA antibody (clone 3F10) at a dilution of 1:250 for HA-DUOX2 or with the anti-FLAG antibody (clone M2) at a dilution of 1:500, at room temperature for 1 h. Cells were subsequently stained with fluorescent secondary antibodies (Alexa488-conjugated goat anti-rat immunoglobulin [Ig]G [H + L] for HA-DUOX2 and Alexa555-conjugated goat anti-mouse IgG [H + L] for DUOX2-FLAG; both from Life Technologies) at a dilution of 1:500 and incubated at room temperature for 30 min. Cells were washed thrice in PBS with 0.05% Tween 20, nuclei were stained with Hoechst 33342, and the stained cells were observed under an FV-1000D confocal microscope (Olympus Corporation, Tokyo, Japan).

### Review meetings

Human phenotype ontology (HPO) tags were assigned to each case based on clinical information sheets filled out by the doctor in-charge of the patient. To narrow down candidate variants, regular review meetings were held for each patient accepted to the TOKAI-IRUD program. Meeting participants included the doctors in-charge of each patient, genetic specialists, and specialists in each disease category, and the discussion focused on patients’ medical histories/phenotypes, the pathogenicity of candidate variants, and reporting of incidental findings.

## Results

### Clinical features of patients

Between 2015 and 2017, a total of 177 patients (81 males; median [range] age, 4 [0–30] years) from 169 families were referred to the TOKAI-IRUD program. All patients registered in this study were new patients, i.e., those who had not been previously analyzed for comprehensive genomic variants; however, several patients have been included in a few subsequent investigations^19–22^.

The TOKAI-IRUD program is open to the possibility of accepting any patient. The clinical symptoms of the applicants were global developmental delay (HP: 0001263; n = 95, 54%), seizures (HP: 0001250; n = 40, 23%), intellectual disability (HP: 0001249; n = 29, 16%), muscular hypotonia (HP: 0001252; n = 24, 14%), dysmorphic facial features (HP: 0001999; n = 17, 9.6%), short stature (HP: 0004322; n = 14, 7.9%), microcephaly (HP: 0000252; n = 11, 6.2%), and others (n = 38, 21%) (Table 1, Supplementary Table S2, and Supplementary Table S3).

**Table 1:** Patient characteristics.

Characteristics	All patients (N = 177)	Diagnostic variants	
		Detected (n = 78)	Not detected (n = 99)
Age at analysis, years, median (range)	4 (0–30)	4 (0–30)	5 (0–24)
**Sex, n (%)**			
Male	81 (46)	34 (44)	47 (47)
Female	96 (54)	44 (56)	52 (53)
**Major symptoms, n (%)**			
Global developmental delay (HP:0001263)	95 (54)	47 (60)	48 (48)
Seizures (HP:0001250)	40 (23)	17 (22)	23 (23)
Intellectual disability (HP:0001249)	29 (16)	13 (17)	16 (16)
Muscular hypotonia (HP:0001252)	24 (14)	16 (21)	8 (8.1)
Dysmorphic facial features (HP:0001999)	17 (9.6)	5 (6.4)	12 (12)
Short stature (HP:0004322)	14 (7.9)	7 (9.0)	7 (7.1)
Microcephaly (HP:0000252)	11 (6.2)	4 (5.1)	7 (7.1)
Others	38 (21)	9 (12)	29 (29)
**Type of samples, n (%)**			
Trio	168 (95)	74 (95)	94 (95)
Duo	9 (5.1)	4 (5.1)	5 (5.1)

### Identified variants

**Figure 1:**
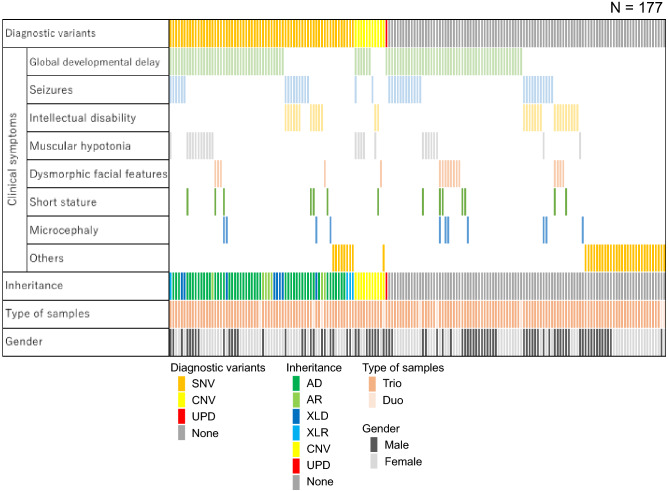
Patient characteristics and information on detected variants. Each column indicates one patient. *SNV* single-nucleotide variant, *CNV* copy number variant, *UPD* uniparental disomy, *AD* autosomal dominant, *AR* autosomal recessive, *XLD* X-linked dominant, *XLR* X-linked recessive.

In accordance with ACMG guidelines, pathogenic SNVs were identified in 36 (20%) patients. Furthermore, 30 (17%) patients carried SNVs classified as “likely pathogenic” based on clinical validity assessment and consistency in clinical information and phenotypes with applicable diseases. Among 66 patients with pathogenic or likely pathogenic SNVs, 47 had autosomal dominant genetic disorders, seven had autosomal recessive genetic disorders, eight had X-linked dominant genetic disorders, and four had X-linked recessive genetic disorders (Fig. 1).

Copy number analysis identified diagnostic duplication/deletion in 11 (6.2%) patients, and these included a 10q26.3 deletion (TOKAI-IRUD-1135 and TOKAI-IRUD-1273), 22q11.2 duplication (TOKAI-IRUD-1236), 5q14.3 deletion (TOKAI-IRUD-1252), 47,XXY (TOKAI-IRUD-1297), 1p36 deletion (TOKAI-IRUD-1301), 7q11.23 duplication (TOKAI-IRUD-1321), 19p13.13 deletion (TOKAI-IRUD-1335), 16p13.3 duplication (TOKAI-IRUD-1337), 17p11.2 duplication (TOKAI-IRUD-1343), and 4p16.3 deletion (TOKAI-IRUD-1475).

ROH analysis identified homozygous regions larger than 10 Mb in 105 cases; this included a diagnostic upd(15)pat in 1 patient (0.6%) who was diagnosed with Angelman syndrome (TOKAI-IRUD-1290, OMIM #105830). Furthermore, UPD of a whole chromosome was identified in 2 (1.1%) patients [upd(2)pat; TOKAI-IRUD-1249 and upd(3)pat; TOKAI-IRUD-1180] with no diagnostic SNVs or CNVs. Thus, genetic diagnoses were obtained for 78 of 177 (44%) patients, and of these, 10 (13%) cases were diagnosed with diseases recognized after 2015, i.e., when this project was initiated. A considerable number of patients showed a milder phenotype (26 [33%]), a more severe phenotype (9 [12%]), or an atypical complex phenotype (17 [22%]) compared to conventional clinical presentation of the respective disease.

### Case presentation of patients with extensive UPD regions

**Figure 2:**
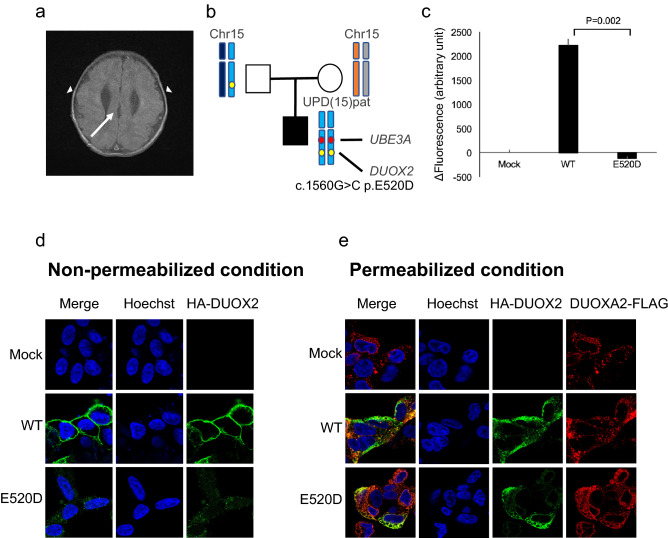
Clinical features and results of UPD analysis of TOKAI-IRUD-1290. **(a)** Brain MRI at the age of 2 years showing cortical dysplasia of the temporal lobes (arrowheads) and corpus callosum dysgenesis (arrow). **(b)** Results of UPD analysis. A paternally inherited UPD region over the entire length of the long arm of chromosome 15 [upd(15)pat] was identified, which covers the region of the UBE3A gene. **(c)** H_2_O_2_-producing capacity of the DUOX2 proteins was measured with Amplex Red reagent in the presence of co-expressed DUOXA2-FLAG. The activity of the mutants were standardized based on those of the WT (100%) and mock-transfected control (0%). Data are representative of three independent experiments (each performed in triplicate) with similar results. T-bars indicate standard errors of the mean.*p < 0 05 vs. WT (Welch’s *t*-test). **(d)** Subcellular localization analysis using HA-tagged DUOX2 constructs (WT or E520D; green fluorescence). **(e)** Fluorescence immunostaining under permeabilized conditions revealed that the localization of E520D-DUOX2 was consistent with DUOXA2.

TOKAI-IRUD-1290 with upd(15)pat: The patient, a 2-year-old boy at the time of sample submission, was the third of three children of healthy non-consanguineous parents (Fig. 2b). Gyrus dysplasia, suspected since the fetal period, was confirmed by magnetic resonance imaging (MRI) after birth (Fig. 2a). He was tube fed due to difficulties with oral intake and a tracheostomy was performed after repeated aspiration pneumonia. He also had congenital hydronephrosis, congenital hypothyroidism, gastroesophageal reflux disease, developmental delay, epilepsy, deafness, and laryngotracheomalacia. ROH analysis identified a paternal UPD region over the entire length of the long arm of chromosome 15 [upd(15)pat], covering the region of the *UBE3A* gene, which led to a diagnosis of Angelman syndrome (OMIM#105830) (Fig. 2b). Additionally, 11 homozygous rare variants were identified in a paternally derived UPD region, which included a *DUOX2* (c.G1560C, p.E520D) variant. *DUOX2* is a known causative gene for congenital hypothyroidism, but this particular variant has not been previously reported.

To verify the pathogenicity of the DUOX2 p.E520D missense substitution detected in this case, expression experiments were conducted using HEK293 cells wherein the H_2_O_2_-producing capacity of the E520D mutant in the presence of co-expressed *DUOXA2*-FLAG was evaluated. We show that the E520D mutant showed complete loss of H_2_O_2_-producing activity (Fig. 2c). Visualization of subcellular localization using immunofluorescence revealed substantial differences in membrane expression levels between the WT and E520D mutant (Fig. 2d,e), indicating that protein localization was affected by the missense substitution.

TOKAI-IRUD-1180 with upd(3)pat: This patient, a 3-year-old girl at the time of sample submission, was the only child of healthy non-consanguineous parents. She suffered seizures beginning on day 1 after birth and symptomatic epilepsy was suspected based on abnormalities detected on an electroencephalogram. However, the seizures ceased from day 14, when oral administration of phenobarbital was initiated. She was unable to sit and had poor language understanding at the time of sample submission. ROH analysis revealed a full-length UPD of chromosome 3 [upd(3)pat], and although 40 homozygous rare missense variants were identified on chromosome 3, it was not possible to arrive at a genetic diagnosis by WES analysis.

TOKAI-IRUD-1249 with upd(2)pat: The patient, a 4-month-old girl at the time of sample submission, was the only child of healthy non-consanguineous parents. A prenatal MRI confirmed hydrocephalus. She was born by scheduled cesarean section at gestational week 34 and suffered from deafness, bilateral club feet, bilateral hip dislocation, multiple joint contractures, congenital hydrocephalus, ventricular septal defect, developmental delay, short and mildly curved femurs, a bell-shaped rib cage, and a vagina without an external opening. ROH analysis revealed a full-length UPD of chromosome 2 [upd(2)pat]. She died of aspiration pneumonia at the age of 10 months, and although 34 rare homozygous missense variants and one nonsense variant were identified on chromosome 2, WES analysis did not lead to a genetic diagnosis.

### Incidental findings

One pathogenic variant of a gene included in the ACMG recommendations for reporting incidental findings was detected in one patient (TOKAI-IRUD-1150), viz, c.C6952T in *BRCA2*. Additionally, discordant parent–child relationships were identified in three families.

## Discussion

Here, we present trio/duo-WES data that correspond to clinical features and diagnoses among patients with previously undiagnosed conditions. A significantly higher proportion of genetic diagnosis was achieved compared to previous reports (44%, 78/177 vs. 24–35%,)^23–26^ (Supplementary Table S1). In addition to the 36 (20%) cases with SNVs that were determined to be pathogenic, 30 (17%) cases were genetically diagnosed as “likely pathogenic SNVs” based on ACMG guidelines along with regular discussions with doctors in-charge, which ensured consistency between relevant SNVs and clinical features and medical histories. Specifically, 20 patients (11%) were found to have variants that met the ACMG guideline PP4 (patient's phenotype or family history is highly specific for a disease with a single genetic etiology), which is a clear indication that in-person interviews with the patient's physician are effective (Supplementary Table S2). Recent advances in sequencing technology have made it possible to analyze not only SNVs but also CNVs and UPDs, using exome sequencing data^2,4^. The cohort of the current study included a significant number of patients with diagnostic CNVs (n = 11) and UPD (n = 1), which may have contributed to the higher proportion diagnoses achieved.

One patient was diagnosed with the Angelman syndrome (TOKAI-IRUD-1290) upon detection of a paternal UPD [upd(15)pat], which contained a paternally derived UBE3A gene locus and 11 rare homozygous variants, including *DUOX2* p.E520D. As the patient had congenital hypothyroidism and *DUOX2* is a known causative gene for congenital hypothyroidism^27^, in vitro experiments were performed with transgenic cells, which confirmed the *DUOX2* E520D variant to be a loss-of-function missense substitution. Hypothyroidism is not considered a common complication of Angelman syndrome, probably because relatively few cases have been reported^28,29^. It is likely that the paternal isodisomy in this patient resulted in simultaneous loss of heterozygosity in the imprinting UBE3A gene and the paternally derived pathogenic *DUOX2* E520D missense substitution, resulting in a complex clinical phenotype, i.e., Angelman syndrome with hypothyroidism.

To date, UPD regions on six chromosomes (6, 7, 11, 14, 15, and 20) are known to contain imprinting genes associated with hereditary diseases^30^; however, the pathological significance of UPDs in other chromosomal regions remains unknown. In this cohort, large areas of UPDs on chromosomes 3 (TOKAI-IRUD-1180) and 2 (TOKAI-IRUD-1249) were detected but their pathogenicity could not be ascertained because there are no known imprinting genes on these chromosomes^30^. While it is possible that rare homozygous variants within UPD regions are responsible for the disease phenotypes in these patients, it was not possible to relate specific variants to corresponding clinical conditions. Nonetheless, similar to the *DUOX2* missense substitution detected in TOKAI-IRUD-1290, it might be possible to diagnose more cases with autosomal recessive diseases in the near future, wherein the pathogenic variants inherited from one parent become homozygous due to isodisomy. Undoubtedly, additional clinical and genetic data are needed to establish the pathogenicity of these large UPD regions with unknown significance.

In summary, WES analysis of 177 patients with undiagnosed conditions resulted in a relatively high genetic diagnostic proportion of 44%, probably due to detailed face-to-face discussions and superior CNV and UPD detection pipelines. The sharing of international clinical and genetic data is expected to further improve the proportion of genetic diagnoses in the near future.

## Supplementary Information

Supplementary Table S1.

Supplementary Table S2.

Supplementary Table S3.

## Data Availability

Sequence data has been deposited at the DNA Data Bank of Japan (DDBJ) Japanese Genotype–phenotype Archive, under accession number JGAS000522.
