# Supplementary material for: Whole-exome analysis of 177 pediatric patients with undiagnosed diseases
Source: Sci Rep. 2022 Aug 26;12:14589. doi: 10.1038/s41598-022-14161-6 (PMC9418234; doi:10.1038/s41598-022-14161-6)
Supplement: Supplementary file 1 — Supplementary Table S1. [file 41598_2022_14161_MOESM1_ESM.docx]

**Supplementary Table 1. Comparison with previous studies**

| References | Patients, n | Patients with diagnostic variants, n (%) | Methods | Type of samples | |
| --- | --- | --- | --- | --- | --- |
|  |  |  |  | Trio, n (%) | Other, n (%) |
| Gahl, *et al*. | 160 | 39 (24) | SNP array, WGS, or WES | Not shown | Not shown |
| Yang, *et al*. | 2000 | 504 (25) | WES | Not shown | Not shown |
| Lee *et al.* | 814 | 213 (26) | WES | 410 (50) | 404 (50) |
| Splinter *et al.* | 382 | 132 (35) | clinical review, clinical testing, SNP array, WGS, or WES | Not shown | Not shown |
| This study | 177 | 78 (44) | WES | 168 (95) | 9 (5) |
